# Intestinal Microbiota Promotes Psoriasis-Like Skin Inflammation by Enhancing Th17 Response

**DOI:** 10.1371/journal.pone.0159539

**Published:** 2016-07-19

**Authors:** Zuzana Zákostelská, Jana Málková, Klára Klimešová, Pavel Rossmann, Michaela Hornová, Iva Novosádová, Zuzana Stehlíková, Martin Kostovčík, Tomáš Hudcovic, Renata Štepánková, Kateřina Jůzlová, Jana Hercogová, Helena Tlaskalová-Hogenová, Miloslav Kverka

**Affiliations:** 1 Institute of Microbiology of the Czech Academy of Sciences, v.v.i., Prague, Czech Republic; 2 Institute of Molecular Genetics of the Czech Academy of Sciences, v.v.i., Prague, Czech Republic; 3 Institute of Microbiology of the Czech Academy of Sciences, v.v.i., Nový Hrádek, Czech Republic; 4 Department of Dermatology, 2nd Medical Faculty, Charles University in Prague and Bulovka Hospital, Prague, Czech Republic; 5 Institute of Experimental Medicine of the Czech Academy of Sciences, v.v.i., Prague, Czech Republic; Ohio State University, UNITED STATES

## Abstract

Psoriasis is a chronic inflammatory skin disease in which Th17 cells play a crucial role. Since indigenous gut microbiota influences the development and reactivity of immune cells, we analyzed the link among microbiota, T cells and the formation of psoriatic lesions in the imiquimod-induced murine model of psoriasis. To explore the role of microbiota, we induced skin inflammation in germ-free (GF), broad-spectrum antibiotic (ATB)-treated or conventional (CV) BALB/c and C57BL/6 mice. We found that both mice reared in GF conditions for several generations and CV mice treated with ATB were more resistant to imiquimod-induced skin inflammation than CV mice. The ATB treatment dramatically changed the diversity of gut bacteria, which remained stable after subsequent imiquimod application; ATB treatment resulted in a substantial increase in the order *Lactobacillales* and a significant decrease in *Coriobacteriales* and *Clostridiales*. Moreover, as compared to CV mice, imiquimod induced a lower degree of local and systemic Th17 activation in both GF and ATB-treated mice. These findings suggest that gut microbiota control imiquimod-induced skin inflammation by altering the T cell response.

## Introduction

Psoriasis is a chronic inflammatory disease that affects approximately 2–4% of the world's population [1]. It is characterized by scaly red plaques of epidermal hyperplasia, dilatation of dermal blood vessels, accumulation of inflammatory cells in dermis and activation of the IL-23/Th17 axis [2]. In genetically predisposed individuals, psoriasis can be initiated by various environmental triggers, including bacterial infection, antibiotic treatment or profound changes in diet [3–5]. These triggers suggest involvement of microbiota in the disease pathogenesis, although the exact molecular mechanisms of this host-microbe interaction are still largely unknown [6].

There is a close association between microbiota and psoriatic attacks [7]. Microbial infections are not only a well-known risk or aggravating factor for psoriasis, but they may even be a tool of natural selection for a pro-inflammatory genotype that favors psoriasis development [8, 9]. The microbiota associated with psoriatic lesions significantly differs from this on healthy skin [10, 11]. But the connection between psoriasis and microbiota may not be limited only to skin microbiota. In murine models of inflammatory diseases, gut microbiota profoundly influenced the immune system development and reactivity [12, 13]. During the early postnatal period, the host-microbe interactions may significantly influence the immune system development and thus change the sensitivity to inflammatory diseases later in life [14]. The outcome of host-microbe interaction could change during the individual's development. So while treatment with antibiotics (mixture of vancomycin and polymyxin B) decreases the severity of psoriasis-like skin inflammation in adult mice, it worsens it in mice born to parents exposed to these antibiotics [15]. Such antibiotic treatment can shift the microbiota composition significantly, although its effect on gut microbiota bacterial load is usually only marginal [16]. Therefore, in this study, we used mice reared in germ-free conditions for several generations to analyze the role of gut microbiota in psoriasis and T cell response as a link between them. Next, we focused on the gut microbiota and analyzed the development of psoriasis-like skin inflammation in conventional mice treated orally with antibiotics.

## Materials & Methods

### Mice

We used female BALB/c or C57BL/6 mice (7–10 weeks old) reared either in conventional or germ-free conditions at the Institute of Microbiology of the CAS. The GF mice were reared in sterile Trexler-type plastic isolators for several generations before they were used in experiments [17]. Mice were fed with Altromin 1414 diet (Altromin, Lage, Germany). All experiments were approved by the Animal Care and Use Committee at the Institute of Microbiology, CAS, approval ID: 050/2011 and 39/2015.

### Murine model of psoriasis

The animals were treated daily for up to 7–8 consecutive days on their shaved back and left ear by either 62.5 mg of imiquimod (IMQ) cream (Aldara, 3M Health Care Limited, Great Britain) or similar amount of control cream (vaseline, Aromatica CZ, Czech Republic). The severity of erythema and scaling was monitored daily by a scale based on the clinical Psoriasis Area and Severity Index (PASI), and ear swelling and skin thickening were measured at the end of the experiment, as described previously [18]. During our preliminary experiments, we found that irradiated IMQ cream (25 kGy), which was used in our experiments, induces skin inflammation of similar nature and degree as the non-irradiated cream (data not shown).

### Antibiotic treatment

Mice were treated with antibiotics 2 weeks before psoriasis induction and the treatment continued until the end of the experiment. A mix of metronidazol (0.4 mg; B. Braun, Melsungen AG, Germany), colistin (0.3 mg) and streptomycin (2 mg) (both Sigma-Aldrich) was administered daily by gavage and vancomycin (0.25 mg/ml; Sigma-Aldrich) was added to autoclaved drinking water. Administration of this ATB mixture by gavage was performed in order to prevent severe dehydration and weight loss, because the mice refused to drink these ATB in their drinking water as described by others [19, 20]. This treatment was well tolerated by all mice and led to significant changes in microbiota composition and to gut phenotype resembling GF animals. To protect the mice from subsequent *Candida* overgrowth, mice were gavaged daily with 20 μg of amphotericin-B (Sigma-Aldrich, St. Louis, MO), starting 3 days before antibiotic treatment until the end of the experiment.

### Histology

The dorsal skin and ear samples were fixed in 5% buffered formalin, dehydrated and embedded in paraffin. Next, 4μm sections were cut and stained with H&E for histopathological examination by an experienced pathologist (P.R.), unaware of the treatment of the mice. The degree of psoriatic skin inflammation was scored on a scale of 0–2 (Table 1).

**Table 1:** Disease severity grading using light microscopy.

Grade	Description
**0.0 (Normal skin)**	• thin epidermis without acanthosis or hyperkeratosis • no inflammatory cellularity or rare single lymphocytes in corium
**0.5 (Minimal changes)**	• discrete scatter of individual lymphocytes in corium
**1.0 (Moderate non-characteristic dermatitis)**	• low-degree thickening, hyperkeratosis and acanthosis of epidermis, but without parakeratosis and accumulation of leucocytes ("microabscesses") in corium • scattered lympho-monocytic infiltrates in corium possibly with sporadic polymorphonuclear leukocytes
**1.5 (Psoriasis-like lesion with incomplete signs)**	• well-expressed hyperkeratosis possibly with focal-discrete parakeratosis • marked irregular acanthosis without prominent "gothic vaults" of rete pegs • diffuse cellular infiltration of corium with focal clustering and scattered polymorphonuclear leukocytes
**2.0 (Severe dermatitis with structures resembling human psoriasis)**	• prominent diffuse hyperkeratosis with salient areas of parakeratosis • scattered focal aggregates of intracorneal polymorphonuclear leukocytes; • dysplasia of epidermis with architectural disarray and nuclear polymorphism, mainly in the basal cell layer • severe acanthosis, apparent "roman and gothic vaults" of rete pegs • prominent, focally massive cellular infiltration of corium with clusters of granulocytes and focal "microabscesses" • congestion of corial capillaries and venules with leukostasis and mural adhesion, edema in tela subcutanea

The degree of imiquimod-induced psoriasis-like skin inflammation was evaluated from histological sections and scored on a scale of 0–2 according the instructions above.

### Flow cytometry

We processed spleen and axillary lymph nodes into single cell suspensions immediately after animals were sacrificed and blocked them as described previously [21]. Then, we performed three different flow cytometry experiments. To analyze major T cell phenotypes, the cells were stained with extracellular fluorochrome-labeled anti-mouse antibodies: PE conjugated anti-CD3 (clone 145-2C11, dilution 1:100) and FITC conjugated anti-γδTCR (clone EbioGL3, dilution 1:50) (all eBioscience, San Diego, CA, USA). To analyze intracellular RORγt, the cells were first stained with FITC conjugated anti-CD3 (clone 145-2C11, dilution 1:100) and then fixed, permeabilized by Intracellular fixation & permeabilization buffer set (eBioscience, San Diego, CA, USA) and stained intracellularly for PE-conjugated anti-RORγt (clone AFJKS-9, dilution 1:50). For intracellular staining of produced cytokines, cells were first stimulated with 1 μg/ml PMA and 2.8 μM Ionomycin (both from Sigma-Aldrich) for 8 hours and then treated with a mixture of Brefeldin A and Monensin (both from eBioscience) for 4 hours, according to the manufacturer's instructions. The cells were stained with extracellular FITC conjugated anti-CD3 (clone 145-2C11, dilution 1:100), fixed, permeabilized and stained for intracellular PE conjugated anti-IL-17 (clone eBio17B7, dilution 1:50) and APC conjugated anti-IFN-γ (clone XMG1.2, dilution 1:50) both from eBioscience. Data were acquired using LSRII (BD Bioscience) and analyzed by FlowJo software v 9.6.2. (Tree Star, Inc., Ashland, OR). Cells that were dead at the time of surface staining were excluded using Fixable viability dye eFluor 780 (dilution 1:200) (eBioscience).

### Cell cultivation and cytokine measurement

Single cell suspensions from spleen and axillary lymph nodes were prepared as described above. The cell viability, generally around 90%, was analyzed using Trypan Blue (Sigma-Aldrich) exclusion and cells were seeded at 2 x 10^5^ of live cells per well in 96-well plates in RPMI 1640 (Sigma-Aldrich) culture medium supplemented with 10% fetal bovine serum (BioClot GmbH, Aidenbach, Germany) and 1% Antibiotic-Antimycotic solution (Sigma-Aldrich). Then, we stimulated the cells for 48 hours with plate-bound anti-CD3 (5 μg/ml; clone 145-2C11) and soluble anti-CD28 (2 μg/ml; clone 37.51; both eBioscience) antibodies. The supernatants were collected and frozen at -20°C until analysis. Commercially available ELISA sets were used to measure the levels of IFN-γ and IL-17 (Invitrogen Corp.) in the supernatants. All tests were performed according to the manufacturers' recommendations.

### Microbiota analysis

Stool samples from ATB-treated and control mice were collected on day 0, 14 (just before psoriasis induction) and 21 (the last day of the experiment). DNA was isolated with MasterPure™ Complete DNA and RNA Purification Kit (Epicentre, Madison, WI, USA).

Next, the V3-V4 region of 16S rRNA gene was amplified using degenerate bacterial 16S rRNA-specific primers 341F (5′-CCTACGGGNGGCWGCAG-3’) and 806R (5′-GGACTACHVGGGTWTCTAAT-3’) which were barcoded to enable multiplexing of sequencing libraries. PCR amplification was performed with KAPA 2G Robust Hot Start DNA Polymerase (Kapa Biosystems, Wilmington, MA, USA), with final concentrations: Buffer B 1x, Enhancer 1x, dNTP 0.2 mM each, primers 0.5 μM each, DNA sample 4 ng/μl, KAPA polymerase 0.5U. Cycle parameters were 3 min 94°C, 25 cycles of 30 s at 94°C, 1 min at 54.2°C, and 1 min 15 s at 72°C, final extension at 72°C for 10 min. Three PCR products were pooled to minimize random PCR bias and the length of PCR product was checked on the agarose gel electrophoresis. Equal amounts of each sample were plate-purified using the SequalPrep™ Normalization Plate (96) Kit (Invitrogen). Equimolar amounts of PCR product from each sample were than pooled and MiSeq platform compatible adapters were ligated using TruSeq DNA PCR-Free LT Kit (Illumina), library was quantified using KAPA Library Quantification Kit (Illumina) and sequenced on MiSeq platform using 2x300bp kit at CEITEC Genomics Core Facility. Sequencing data were processed using QIIME (Quantitative Insights Into Microbial Ecology) version 1.9.1 [22]. The sequence data are available in the Sequence Read Archive (SRA) http://www.ncbi.nlm.nih.gov/sra under the accession number SRP068451. To measure the bacterial load, extracted DNA was analyzed by recently optimized qPCR assay, using universal bacterial primer pair [23]

### Statistical analysis

Unpaired Student's t-test was used to compare two experimental groups and one-way analysis of variance (ANOVA) with Dunnett's multiple comparison test was used to compare the multiple experimental groups with the control group. The data are presented as the mean ± standard deviation (SD), unless stated otherwise, and differences are considered statistically significant at P ≤ 0.05. The GraphPad Prism software (version 5.0, GraphPad Software, Inc., La Jolla, CA, USA) was used for all statistical analyses.

## Results

### Imiquimod induces milder skin inflammation in germ-free than in conventional mice

**Fig 1:**
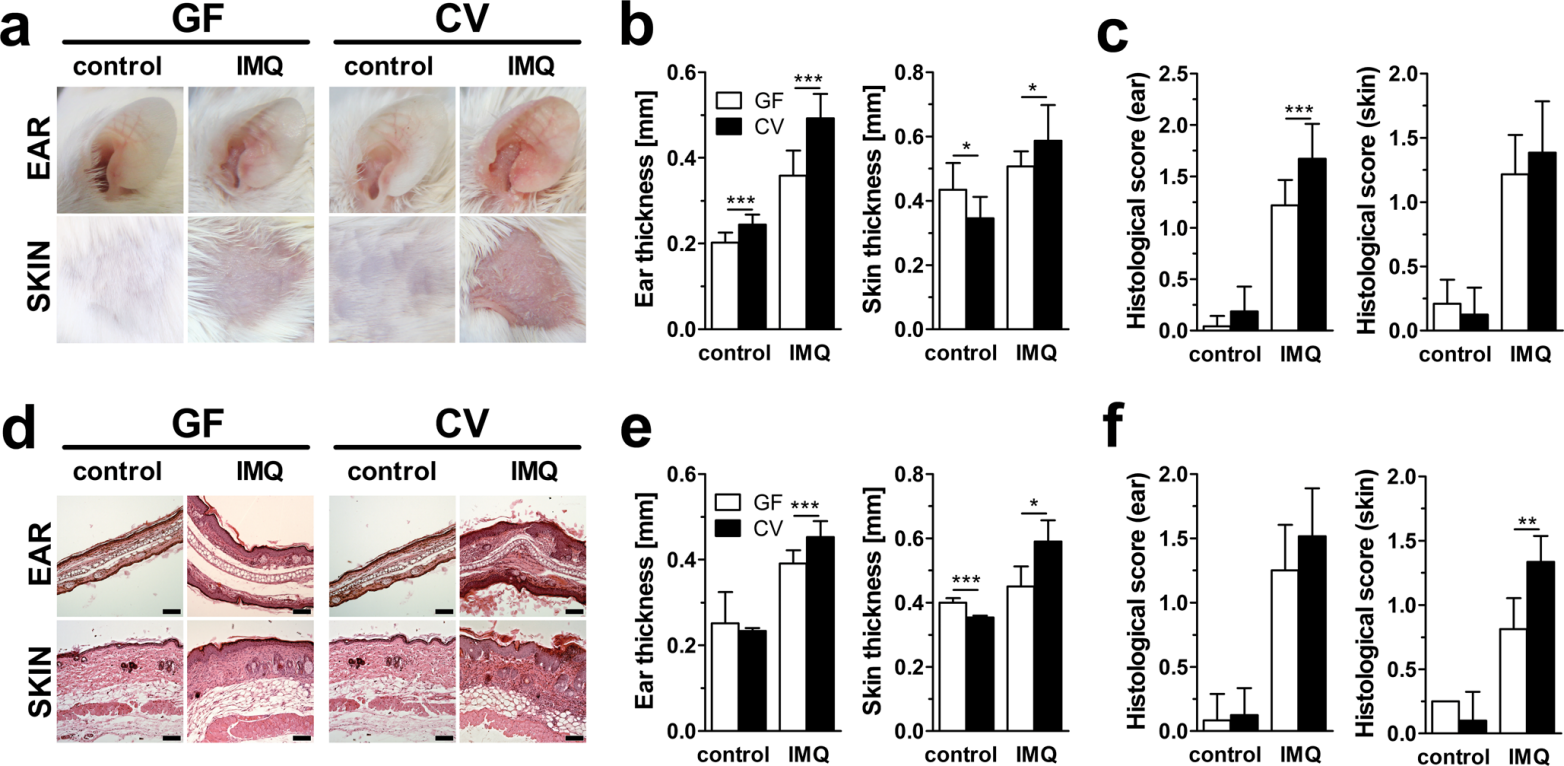
Microbiota enhances sensitivity to IMQ-induced skin inflammation in BALB/c and C57BL/6 mice. (a) Representative macroscopic pictures of healthy (control) and inflamed (IMQ) ear or dorsal skin of BALB/c mice, illustrating the disease severity. Quantification of skin and ear thickness of BALB/c (b) and C57BL/6 (e) mice. Quantification of histopathological score (0–2) after H&E staining of the ear and skin of BALB/c (c) a C57BL/6 (f) mice. (d) Representative H&E-stained ear and skin sections of BALB/c mice (scale bar, 100 μm). The values represent means ± SD as a pool of three independent experiments (n = 10–20 mice per group). *p < 0.05, ***p < 0.001.

To analyze the role of microbiota in the murine model of psoriasis, we treated ear and shaven dorsal skin of germ-free (GF) and conventionally reared (CV) BALB/c mice with imiquimod (IMQ). Control mice were treated with control cream in a similar manner. We have not observed any differences in skin morphology between GF and CV mice. While IMQ-treated mice developed typical signs of psoriasis, such as redness, scaling and increased epidermal thickening, skin of the control mice manifested neither any signs of macroscopic nor microscopic inflammation (Fig 1). All changes found in shaved dorsal skin were usually mirrored by these found in ear skin. As compared to CV mice, GF mice displayed less severe erythema and scaling (Fig 1A), less severe ear and skin thickening (Fig 1B) and less severe hyperkeratosis, acanthosis and leukocyte infiltration into the dermis (Fig 1C and 1D). Moreover, while quite common in skin of CV mice, we did not find any parakeratosis, acanthosis with prominent gothic vaults or focal microabscesses in the dorsal or ear skin of GF mice (Fig 1D).

As reported by others, genetic background of mice may influence the development of IMQ-induced skin inflammation, with BALB/c being more sensitive and C57BL/6 more resistant to disease induction [18]. To overcome this issue, we performed a similar experiment also in C57BL/6 mice. Although the disease severity differed slightly between C57BL/6 (Fig 1E and 1F) and BALB/c (Fig 1A, 1B, 1C and 1D) mice, both GF strains were still more resistant to IMQ-induced skin inflammation than CV. These results show that microbiota aggravates the disease development leading to psoriasis, regardless of genetic background.

### Antibiotics change gut microbiota composition and prevent severe forms of skin inflammation

**Fig 2:**
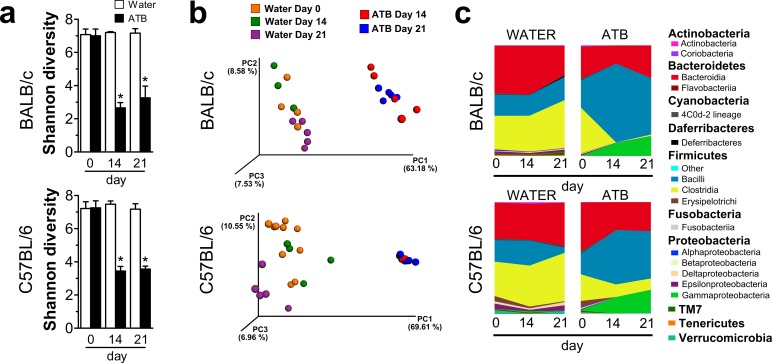
Antibiotic treatment changes the microbiota composition in both BALB/c and C57BL/6 mice. (a) Comparison of diversity in microbiota between the conventional (CV) and ATB-treated mice using Shannon diversity index. (b) Principal coordinates analysis (PCoA) plot using the unweighted UniFrac distance metric shows the compositional similarity before and after ATB treatment (Day 0, Day 14, Day 21). Each colored orb represents the microbiota composition in feces of one mouse. Each color represents each group of mice at the day 0, 14, 21. (c) The microbial composition in time is displayed as mountain plot before (Day 0) and after ATB treatment (Day 14), and after the induction of skin inflammation (Day 21) in comparison to CV mice. The figure shows (a) means ± SD or (c) means from pool of 5 mice. Statistical significance was determined by unpaired Student t test; *p < 0.05, **p < 0.01.

Microbiota significantly influences the immune system reactivity, and even the early germ-free period may have a major impact on the sensitivity to diseases later in life [14]. To analyze if the disease development can be altered by microbiota reduction in adult mice, we treated mice with a broad-spectrum antibiotic mixture (ATB), starting two weeks before the IMQ treatment and continuing until the end of the experiment. To analyze the effect of this antibiotic treatment on microbial load and composition, we performed 16S rRNA-based qPCR and next generation sequencing, respectively. We collected samples of feces before the ATB treatment (Day 0), before the imiquimod application (Day 14), and at the end of the experiment (Day 21). We found that oral ATB dramatically decreased the gut microbial diversity (Fig 2A) and shifted its composition (Fig 2B). This treatment, however, did not change the microbial load; because in 1g of stool there were 8.4 ± 4.9 x 10^10^, 6.2 ± 3.7 x 10^10^ and 7.1 ± 1.2 x 10^10^ (mean ± SD) copies of eubacterial 16S gene at day 0, 14 and 21, respectively. There were no significant changes in microbiota due to the IMQ treatment alone (Fig 2B and 2C). After ATB treatment, we found a substantial increase in the proportion of phylum *Firmicutes* which was caused by an increase in order *Lactobacillales* even though other members of the same phylum, such as *Clostridiales* and *Erysipelotrichiales*, were decreased (Fig 2C). On the other hand, members of orders *Coriobacteriales* and *Campylobacterales* (Fig 2C) were both significantly decreased after ATB treatment. We found that ATB had a similar effect on microbiota in both strains of mice, except for significant increase of *Enterobacteriales*, which was apparent in C57BL/6 but not in BALB/c mice. There were no significant changes in microbiota due to the IMQ treatment (Fig 2B and 2C).

**Fig 3:**
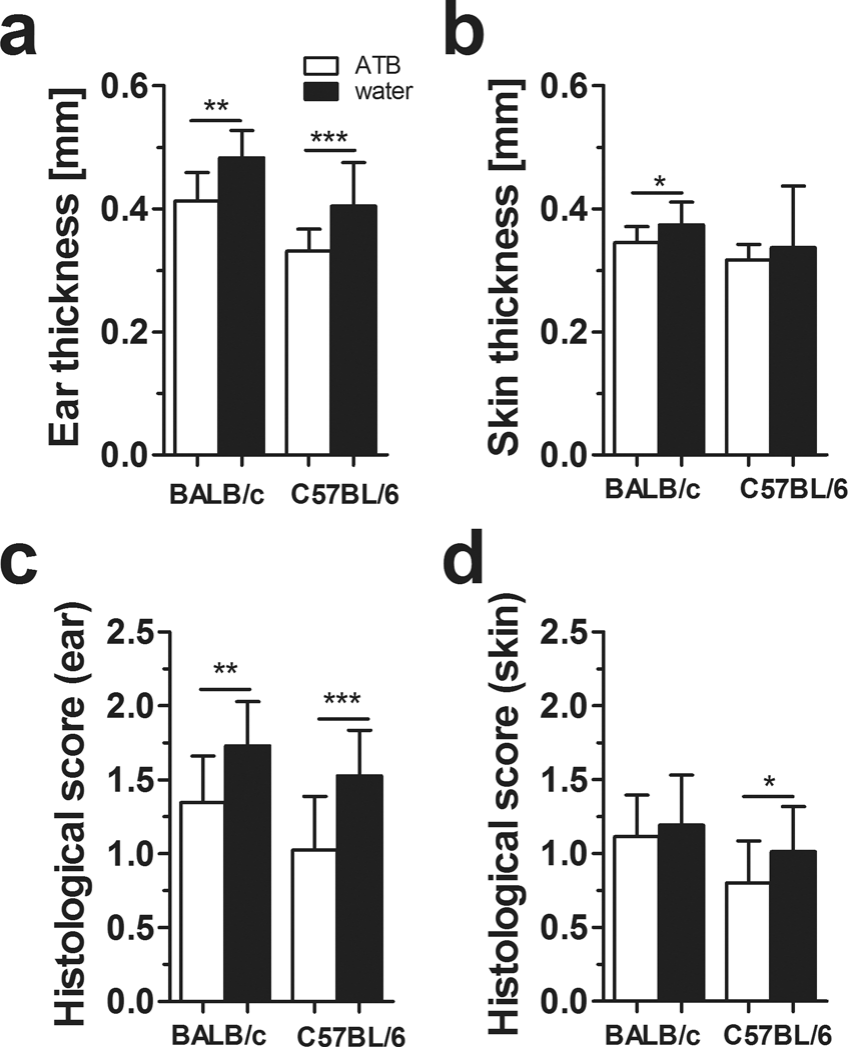
Antibiotic treatment decreases the skin inflammation in both BALB/c and C57BL/6 mice. Quantification of ear (a) and skin (b) thickness. Quantification of histopathological score (0–2) after H&E staining of the ear (c) a skin (d). The graphs show 18–19 mice per group (a pool of three independent experiments). Statistical significance was determined by unpaired Student t test; *p < 0.05, **p < 0.01.

We found that IMQ induced similar inflammatory changes in mouse skin, including edema (Fig 3A and 3B), erythema, scaling (data not shown) and histological changes (Fig 3C and 3D) in all treated groups but the degree of these changes was significantly lower in both strains of ATB-treated animals as compared to their respective controls.

### Microbiota drives the skin inflammation by inducing stronger T cells response

**Fig 4:**
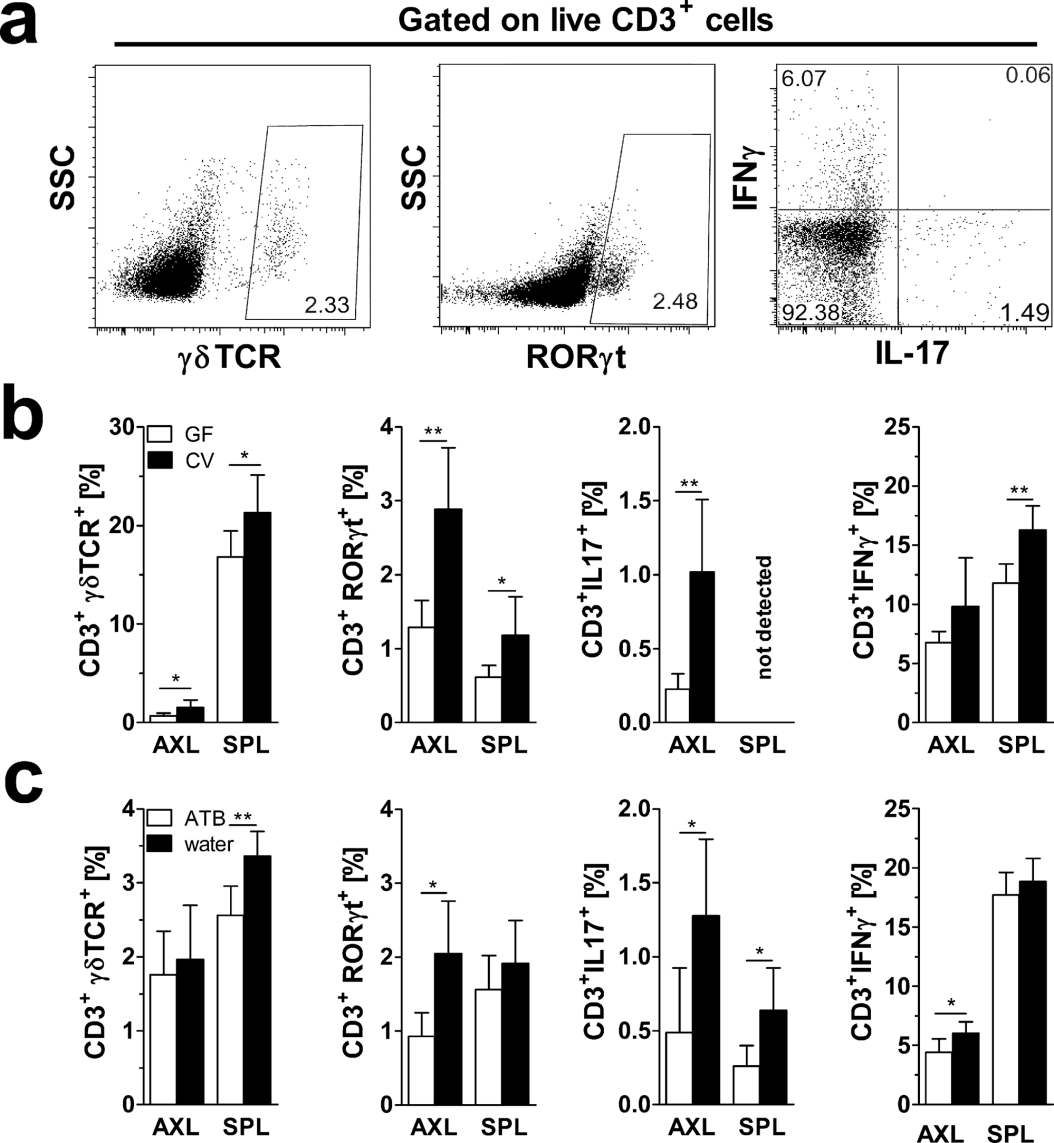
Absence of microbiota or ATB treatment decreases the percentage of γδ T cells and Th17 cells in spleen or axillary lymph nodes of IMQ-treated mice. Cells from spleen or axillary lymph nodes were isolated and either analyzed for surface γδTCR or intracellular RORγt or stimulated *in vitro* for 8 h by PMA and Ionomycin, the last 4h in the presence of Brefeldin A and Monensin, and analyzed for intracellular IL-17A and IFNγ production by flow cytometry. Cells were first gated for live cells and CD3^+^ and subsequently on γδTCR^+^, RORγt^+^ or IL-17^+^ and INFγ^+^ cells as shown on the (a) example of gating strategy. These results from one representative experiment (n = 5–8 mice per group) out of three independent experiments with (b) GF versus CV mice or with (c) ATB-treated versus control mice are quantified in the graphs. Statistical significance was determined by unpaired Student t test; *p < 0.05, **p < 0.01.

Microbiota has a major impact on T-cell development and both psoriasis and its murine model result in significant local accumulation of γδTCR^+^ and Th17 cells [12, 24, 25]. Therefore, we evaluated how microbiota influences the frequency of γδTCR^+^ T cells and Th17 cells, both locally (in draining lymph nodes) and systemically (in spleen). We found that, in general, mice with reduced or absent microbiota have significantly lower frequencies of these T cells both locally and systemically, although this effect is slightly less pronounced in ATB-treated mice as compared to GF mice (Fig 4). In both groups, there is even an indication of a lower number of IFN-γ^+^ T cells in GF and ATB-treated mice, but these were only significant in spleen of GF and axial lymph nodes of ATB-treated mice (Fig 4B and 4C).

**Fig 5:**
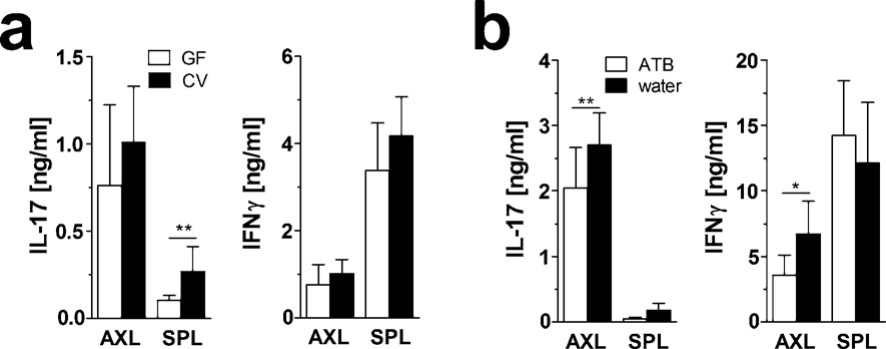
Absence of microbiota or ATB treatment reduces the production of pro-inflammatory cytokines by T cells from IMQ-treated mice. Cells isolated from spleen or axillary lymph nodes of (a) GF and CV mice or (b) ATB-treated and control mice were stimulated for 48hours *in vitro* by plate-bound anti-CD3 antibody and soluble anti-CD28 antibody. Cell culture supernatants were analyzed for IL-17A and INF-γ by ELISA. These data are representative of three independent experiments (n = 5–8 mice per group) with similar results. Statistical significance was determined by unpaired Student t test; *p < 0.05, **p < 0.01.

Another consequence of IMQ application is splenomegaly and activation of the IL17/IL-23 axis which leads in increased IL-17 and INF-γ production [18]. In our experiments, IMQ induced splenomegaly in CV mice which was less prominent in GF and ATB-treated mice (data not shown). To analyze dominant T cell response in lymphatic organs of IMQ-treated mice, we stimulated cells from their draining (axial) lymph nodes or spleens with anti-CD3 and anti-CD28 antibodies. We found that cells from CV mice produced more IL-17 than those from GF mice (Fig 5A). Moreover, IL-17 production in cells from axial lymph nodes was also higher in CV mice than in ATB-treated mice (Fig 5B). This is consistent with the high proportion of RORγt^+^ and IL-17^+^ cells we found in CV mice by flow cytometry (Fig 4C). We found a similar trend also for INF-γ production, but its production was always higher in spleen cells than in cells from axial lymph nodes (Fig 5A and 5B).

## Discussion

Continuous interactions between microbiota and the immune system are important for the establishment and maintenance of host homeostasis. Thus change in microbiota composition could lead to a shift in immune system reactivity and ultimately to inflammatory diseases [26]. Here, we analyzed the role of microbiota in the early stages of psoriatic plaque formation using a murine model of psoriasis–IMQ-induced skin inflammation. We found that GF mice have significantly milder skin inflammation than CV mice. Unlike the ATB-treated animals, GF mice did not have any contact with live bacteria, because they were reared in germ-free condition for several generations. This is important, because even prenatal exposure to microbes permanently changes the immune system reactivity [27]. Although mice develop skin inflammation that closely resembles plaque-type psoriasis in humans, different murine strains may display specific disease characteristics [18, 28]. Therefore, we performed our experiments using two different mouse strains, each with distinct immune system reactivity. Nevertheless, the microbiota changes had a similar impact on the disease severity in both strains of mice. This suggests that microbiota influences mechanisms of skin inflammation that are not related to the genetic differences between these two strains.

There is emerging evidence supporting the existence of communication axes between organs, such as gut-skin axis [29, 30]. For example, atopic dermatitis or rosacea are both associated with marked changes in gut barrier and in intestinal microbiota [31, 32], suggesting that not only skin microbiota influences the disease pathogenesis. Altered gut microbiota (dysbiosis) is a hallmark of chronic gastrointestinal diseases, such as inflammatory bowel disease or celiac disease, which are often associated with skin inflammation [33, 34]. Interestingly, gut dysbiosis similar to this in inflammatory bowel disease was found in patients with psoriatic arthritis [35]. Moreover, gut dysbiosis is a common feature also in patients with rosacea, and treatment with oral nonabsorbed antibiotic markedly improves skin inflammation [36].

Since the GF animals lack microbiota both on skin and in gut throughout their life, we analyzed the microbiota-gut-skin axis by changing the gut microbial ecology in CV mice just before psoriasis induction using oral broad-spectrum ATB. We found that, similarly as in GF mice, ATB-treated animals have significantly milder skin inflammation than CV mice. These results are in agreement with a recently published study using even less complex antibiotic mixture of vancomycin and polymyxin B [15]. Therefore, this protective effect is not limited to the complete absence of microbiota during the early postnatal period and the skin inflammation severity could be modified by targeting gut microbiota in adult animals. Interestingly, even though the antibiotic regime used in our experiments was very harsh, it was not able to kill all gut bacteria and the resistant species quickly filled the vacated niche. This newly established ecosystem was, however, markedly different. We found that ATB treatment led to an increase in Firmicutes caused mainly by a massive increase of the *Lactobacilalles*, even though other members of this phylum, such as *Clostridialles* and *Erysipelotrichales*, were decreased. The potential protective role of lactobacilli in skin diseases is supported by findings of decreased intestinal lactobacilli in children with atopic dermatitis and by beneficial effect of their oral administration [37–39]. This immuno-modulatory effect of the gut lactobacilli can be mediated through their ability to suppress the IL-23/Th17 axis [40], which is intimately linked to the pathogenesis of psoriasis. The increase of these anti-inflammatory lactobacilli may then push this delicate balance back towards an anti-inflammatory phenotype.

To analyze the impact of gut microbiota on the IL-23/Th17 axis, we measured the Th17 and γδTCR-bearing lymphocytes in draining lymph nodes and in spleen of GF, ATB-treated and CV mice. We found that GF mice, and to a lesser extent also ATB-treated mice, had lower numbers of both γδTCR^+^ cells and Th17 cells as compared to CV mice. These data suggest that the absence of microbiota, or its change due to the ATB treatment, decreases the pro-inflammatory T cell response and thus decreases the severity of IMQ-induced skin inflammation. This is further supported by other studies describing the ability of commensal bacteria to modulate T cell development [12, 17]. The importance of this connection for psoriasis is still poorly understood, but a molecular link between microbe-dependent Th17 development and psoriasis was recently suggested [41, 42].

Taken together, our results suggest that host interactions with live microbes, possibly from the orders *Clostridiales* and *Erysipelotrichales*, are involved in the pathogenesis of IMQ-induced skin inflammation by influencing the Th17 cell reactivity. The positive effect of gut microbiota modulation by antibiotics on the severity of skin inflammation suggests the involvement of gut-skin axis and may represent the groundwork for novel approaches in psoriatic patient's management.
